# Intramolecular and Intermolecular Interactions of Protein Kinase B Define Its Activation In Vivo

**DOI:** 10.1371/journal.pbio.0050095

**Published:** 2007-04-03

**Authors:** Véronique Calleja, Damien Alcor, Michel Laguerre, Jongsun Park, Borivoj Vojnovic, Brian A Hemmings, Julian Downward, Peter J Parker, Banafshé Larijani

**Affiliations:** 1 Cell Biophysics Laboratory, Lincoln's Inn Fields Laboratories, London Research Institute, Cancer Research UK, London, United Kingdom; 2 Institut Européen de Chimie et Biologie, Pessac Cedex, France; 3 Friedrich Miescher Institute for Biomedical Research, Basel, Switzerland; 4 Advanced Technology Development Group, Gray Cancer Institute, Mount Vernon Hospital, Northwood, United Kingdom; 5 Signal Transduction Laboratory, Lincoln's Inn Fields Laboratories, London Research Institute, Cancer Research UK, London, United Kingdom; 6 Protein Phosphorylation Laboratory, Lincoln's Inn Fields Laboratories, London Research Institute, Cancer Research UK, London, United Kingdom; University of California, United States of America

## Abstract

Protein kinase B (PKB/Akt) is a pivotal regulator of diverse metabolic, phenotypic, and antiapoptotic cellular controls and has been shown to be a key player in cancer progression. Here, using fluorescent reporters, we shown in cells that, contrary to in vitro analyses, 3-phosphoinositide–dependent protein kinase 1 (PDK1) is complexed to its substrate, PKB. The use of Förster resonance energy transfer detected by both frequency domain and two-photon time domain fluorescence lifetime imaging microscopy has lead to novel in vivo findings. The preactivation complex of PKB and PDK1 is maintained in an inactive state through a PKB intramolecular interaction between its pleckstrin homology (PH) and kinase domains, in a “PH-in” conformer. This domain–domain interaction prevents the PKB activation loop from being phosphorylated by PDK1. The interactive regions for this intramolecular PKB interaction were predicted through molecular modeling and tested through mutagenesis, supporting the derived model. Physiologically, agonist-induced phosphorylation of PKB by PDK1 occurs coincident to plasma membrane recruitment, and we further shown here that this process is associated with a conformational change in PKB at the membrane, producing a “PH-out” conformer and enabling PDK1 access the activation loop. The active, phosphorylated, “PH-out” conformer can dissociate from the membrane and retain this conformation to phosphorylate substrates distal to the membrane. These in vivo studies provide a new model for the mechanism of activation of PKB. This study takes a crucial widely studied regulator (physiology and pathology) and addresses the fundamental question of the dynamic in vivo behaviour of PKB with a detailed molecular mechanism. This has important implications not only in extending our understanding of this oncogenic protein kinase but also in opening up distinct opportunities for therapeutic intervention.

## Author Summary

Regulation of intracellular signaling depends on the precise operation of molecular switches such as kinases and phosphatases. Disruption of their activities leads to inappropriate cellular proliferation, growth, and survival. Protein kinase B (PKB) is a critical kinase that regulates events downstream of growth factor receptors. It is also involved in human cancer, where its overexpression induces malignant transformation. We studied the molecular mechanisms of PKB's interaction with its upstream regulator, 3-phosphoinositide–dependent protein kinase 1 (PDK1). By using a fluorescent probe, we monitored the conformational changes of PKB in cells using Förster resonance energy transfer detected by fluorescence lifetime imaging microscopy. Applying this approach, we show that PKB and PDK1 are found as complexes in the cytoplasm. Despite this proximity to its regulator, we show that PKB remains inactive through an intramolecular interaction of its pleckstrin homology (PH) domain and kinase domain. We refer to this inactive state as the “PH-in” conformer. Following growth factor activation, PKB changes conformation to the “PH-out” conformer and is phosphorylated by PDK1. The active “PH-out” conformer dissociates from the plasma membrane to phosphorylate downstream proteins. Our in vivo studies provide a new model for the mechanism of PKB.

## Abstract 3

In vivo experiments detail how PKB can switch from an inactive "PH-in" state (with intramolecular interactions between the PH and kinase domains) to an active "PH-out" state coincident with plasma membrane recruitment.

## Introduction

A key downstream relay in various growth factors and hormones is the activation of the serine/threonine protein kinase (PKB/Akt). PKB activates a plethora of proteins that are involved in metabolism, proliferation, growth, and survival [1–3]. Several lines of evidence indicate that the PKB pathway is involved in human cancer, and in particular, its overexpression induces malignant transformation and chemoresistance [2,4,5]. Its activation is thought to proceed through the recruitment of the protein to membranes via interaction of its PH domain with the phosphoinositides produced by phosphoinositide-3-kinase [specifically PtdIns (3,4,5)P_3_ and PtdIns (3,4)P_2_] [6,7]. The lipid-bound PKB is then phosphorylated by 3-phosphoinositide–dependent protein kinase 1 (PDK1), which is also recruited through its PH domain binding to PtdIns (3,4,5)P_3_. The PDK1 phosphorylation, critical for activation, occurs at Thr308 in the activation T-loop of PKBα [8]. A second phosphorylation within a C-terminal hydrophobic motif at Ser473 acts in synergy to fully activate the protein kinase. It is believed that this phosphorylation occurs via the mTor:rictor pathway [9]. Our understanding of the mechanism of activation of PKB remains limited as, unlike other AGC kinases that are substrates for PDK1 [10], direct in vitro or in vivo interaction of PKB with PDK1 has not been observed. The current models of PKB activation only speculate, based on the regulation of other AGC kinases, how PKB may change its conformation to interact with PtdIns at the plasma membrane and thereafter be activated by PDK1. The functionally independent behaviour of the PH and kinase domains of PKB is well characterised; the structure of both has been solved separately [6,7,11–13]. In isolation, the PH domain retains intrinsic lipid binding properties and, similarly, the kinase domain retains function [14,15]. The kinase domain is well understood structurally with respect to its inactive and active conformers [12,13]. However, the more complex interactions between the PH and kinase domains have not succumbed to direct structural analysis and remain to be elucidated. The recent advent of an in vivo probe to monitor PKB conformational changes in conjunction with molecular modeling has permitted analysis of the mechanism by which these two domains interact and how PKB changes conformation in relation to its activation by PDK1. We have addressed the possible molecular mechanisms involved in the conformational dynamics of PKB and dissected its activation in intact cells. This has important implications not only in extending our understanding of this critical regulator but also in opening distinct opportunities for therapeutic intervention.

## Results

### PDK1 Precomplexes with Its Substrate PKB in the Cytoplasm

**Figure 1:**
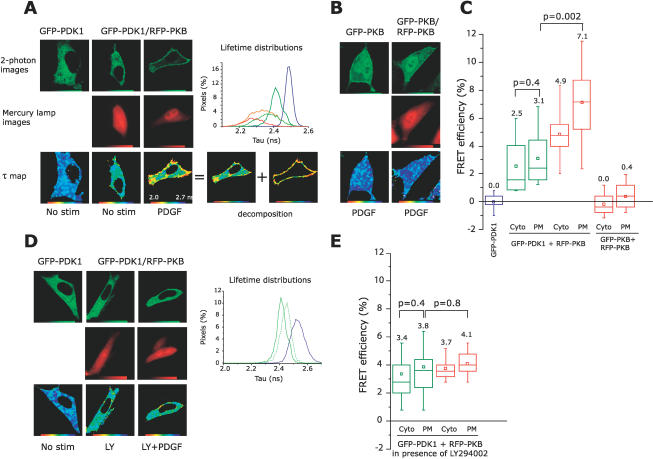
PDK1 Interacts with PKB in a Phosphatidylinositol 3-Kinase–Dependent Manner at the Membrane and in a Phosphoinositide-Independent Manner in the Cytoplasm (A) NIH3T3 cells were transfected with GFP-PDK1 alone or with RFP-PKB. The lifetimes are presented in pseudo-colour scale ranging from 2.0 to 2.7 ns. The graph presents the lifetime distributions of the cells for the following conditions: GFP-PDK1 (blue), GFP-PDK1 with RFP-PKB prior (green) and upon PDGF stimulation (orange). The red and the light green lines indicate the lifetime distributions of the plasma membrane and cytoplasm pixels, respectively, after separation of the compartmental pixels (see decomposition). (B) NIH3T3 cells were transfected either with GFP-PKB alone or together with RFP-PKB. The FRET efficiency variation is statistically insignificant. (C) The statistical analysis of experiments in A and B are presented as box and whiskers plots (see Supporting Information). The FRET efficiency was calculated for at least ten cells per condition for the cytoplasmic (Cyto) and plasma membrane (PM) components, as indicated. The first column shows the variation of the donor lifetime (blue) centred at zero. The dispersion is less than ±1%. The FRET efficiency of GFP-PDK1 + RFP-PKB condition is presented before (green boxes) and after (red boxes) PDGF stimulation. The FRET efficiency of the control GFP-PKB + RFP-PKB upon PDGF is presented by the two last boxes. The mean value of the FRET efficiency is indicated on top of each box. The highly significant FRET efficiency variations at (PM), for experiment A, is indicated by a *p-*value of 0.002 (see Supporting Information). (D) Similar experiment as in (A) was performed but cells were first pretreated for 20 min with LY294002. The graph shows the superposition of the lifetime distributions of coexpressed GFP-PDK1 and RFP-PKB pretreated with LY294002 alone (green line) or pretreated with LY294002 before PDGF stimulation (dashed green line). Control GFP-PDK1 lifetime distribution is shown in blue. (E) The FRET efficiency between LY294002- and LY294002 + PDGF–treated cells do not vary significantly (*p* = 0.8).

It has been assumed that upon activation of various growth factor pathways, PDK1 and PKB uniquely colocalise at the plasma membrane, permitting PDK1 to phosphorylate PKB. To assess in single cells the dynamic relationship between PKBα and PDK1, Förster resonance energy transfer (FRET) was exploited. Two fusion proteins were generated by genetically encoding enhanced green fluorescent protein (EGFP) (donor) at the N terminus of PDK1 and monomeric RFP (acceptor) at the N terminus of PKBα. NIH3T3 cells were transfected with the expression vectors for GFP-PDK1 and RFP-PKB, and the expressed proteins behaviours were followed upon stimulation of the platelet-derived growth factor (PDGF) receptor pathway (note that the levels of expression were the minimum required for data acquisition). For optimum spatial resolution, FRET was monitored by two-photon fluorescence lifetime imaging microscopy (FLIM). Resonance energy transfer was detected by the decrease in the donor lifetime in the presence of the acceptor. The variations in lifetime are presented by lifetime distributions curves and mean FRET efficiency. Coexpression of RFP-PKB with GFP-PDK1 in NIH3T3 cells (Figure 1A) induced a decrease in the lifetime of GFP-PDK1. The decrease in the donor lifetime is quantitatively presented by the lifetime distribution curves. The blue curve is the GFP-PDK1 when expressed alone, and the green curve is when it is expressed with RFP-PKB (acceptor). From this reduction in lifetime, we deduced that, at steady state, GFP-PDK1 and RFP-PKB interact in the cytoplasm. The cytoplasmic mean FRET efficiency presented as box and whiskers plots is 2.5 (Figure 1C). Upon PDGF stimulation, both PKB and PDK1 translocated to the plasma membrane, and a further decrease in the average lifetime was detected (orange curve). The translocation of RFP-PKB to the plasma membrane is clearly shown in Figure S6. The separation of the lifetime distributions at the plasma membrane versus the cytoplasm (red curve: plasma membrane; light green curve: cytoplasm) showed that at the plasma membrane, the mean FRET efficiency is 7.1, and in the cytoplasm, it is 4.9 (Figure 1C). These results indicated that PDK1 and PKB interacted under basal conditions and that the stimulation of the PDGF receptor pathway promoted an enrichment of the PKB–PDK1 complex at the plasma membrane. This intermolecular interaction was also demonstrated by frequency domain FLIM (Figure S1). This behaviour indicates that in the cytoplasm, the PKB–PDK1 complex is at equilibrium determined by the bulk phase concentrations. Upon stimulation, the local concentration of both proteins is enhanced at the plasma membrane, whereby the equilibrium is shifted toward the formation of the PKB–PDK1 complex.

To verify that the PKB–PDK1 interaction was due to docking and not purely concentration effects in the cytoplasm due to transfection, GFP-PKB alone or together with RFP-PKB was expressed in NIH3T3 cells (Figure 1B). The lifetimes from cells with expression levels of donor (GFP-PKB) and acceptor (RFP-PKB), comparable to those of GFP-PDK1 and RFP-PKB, were identified. Despite the colocalisation of GFP-PKB and RFP-PKB in the cytoplasm and the corecruitment of both PKBs at the plasma membrane, upon activation, the FRET efficiency did not change. This is clearly illustrated by the complete lack of lifetime variation (Figure 1B and 1C). These data indicated that PDK1 and PKB were docking in the cytoplasm and at the plasma membrane under steady state and stimulated conditions.

To further investigate whether the docking was PtdIns (3,4,5)P_3_ dependent, cells were pretreated with the phosphatidylinositol 3-kinase inhibitor LY294002 prior to PDGF stimulation. Figure 1D shows that with LY294002, the translocation of PKB and PDK1 is prevented. However, a cytoplasmic FRET efficiency of 3.4, similar to basal conditions, was still observed (Figure 1E). The PDGF-induced phosphorylation of Thr308 and Ser473 was reduced on pretreatment with LY294002 (Figure S2E). This indicated that the recruitment of the PKB–PDK1 complex to the plasma membrane was dependent on PtdIns (3,4,5)P_3_ production, while the docking of PKB to PDK1 in the cytoplasm was PtdIns (3,4,5)P_3_ independent. To assess the role of PtdIns (3,4,5)P_3_ in an inhibitor-independent manner, PKB and PDK1 PH domain mutants (mutants that do not efficiently bind to phosphoinositide lipids: PDK1 RRR and PKB R25C [16,17]) were used to determine their influence on interaction with their wild-type binding partners. Cells were cotransfected with GFP-PDK1/RFP-PKB R25C or GFP-PDK1 RRR/RFP-PKB. Under basal conditions, both wild-type proteins interacted with their mutant partner (Figure S3A and S3B). However, upon activation, the PH mutants did not translocate to the plasma membrane and a change in FRET efficiency was not detected (Figure S3C). It is concluded that complex formation in the cytosol is independent of lipid binding.

PDK1 docks with phosphorylated/acidic hydrophobic motifs at the C termini of some AGC kinases. This is affected through a PDK1 interacting fragment domain (PIF pocket) and additionally through a phosphate-binding site within the upper lobe of the PDK1 kinase domain. PDK1 mutants defective in these interactions, namely the L155E mutant in the PIF pocket and the R131A mutant in the phosphate-binding pocket, still interacted with PKB in the cytoplasm or on PDGF stimulation at the plasma membrane (Figure S4A and S4B). Moreover, the PKB mutant that is not phosphorylated in response to agonists, T308A/S473A (Figure S4C), also retained binding capacity, confirming independently that PKB Ser473 phosphorylation was not required for docking into PDK1′s phosphate-binding pocket. Therefore, the interaction of PKB and PDK1 is independent of the PIF and the phosphate-binding pockets and does not require phosphorylation of Thr308 or Ser473 (Figure S4D). In line with this observation, it has also been shown by using knock-in mutants of PDK1 that the PIF pocket and the phosphate-binding pocket of PDK1 were not implicated in the activation of PKB [18,19]. Therefore, the interaction in the cytoplasm occurs via mechanisms that are distinct from those of other AGC kinases.

Since these kinases interacted in the cytoplasm, it was of interest to determine why PKB was not constitutively phosphorylated by the associated PDK1 in the basal state. One of the mechanisms that regulate the basal levels of Ser473 is the dephosphorylation of this residue by a serine/threonine protein phosphatase, PP2A, implicated in the downregulation of PKB activation [20]. It was postulated that dephosphorylation may dominate the cytoplasmic phosphorylation of PKB. To test this hypothesis, PP2A activity was blocked by treating cells with okadaic acid. The time-dependent increase of RFP-PKB Ser473 phosphorylation upon treatment indicated that in the absence of PP2A, phosphorylation of Ser473 was enhanced, showing that in NIH3T3 cells PP2A regulates Ser473 phosphorylation in the cytoplasm (Figure S2A). The okadaic acid–insensitive phosphatase PHLPP [21] cannot therefore regulate the basal phosphorylation process. Unlike Ser473, the okadaic acid stimulation of Thr308 was modest. It was possible that the “basal state” phosphorylation induced by okadaic acid occurred via a transient PtdIns (3,4,5)P_3_–associated form. To investigate this, we exploited the nonbinding PH domain mutant of PKB (RFP-PKB R25C). The okadaic acid–induced phosphorylation of Ser473 and Thr308 did not differ significantly from that of wild-type PKB, whereas the PDGF-induced phosphorylation of RFP-PKB R25C was significantly reduced (Figure S2B). These data indicated that the okadaic acid–induced phosphorylations occurred in the cytoplasm. In RFP-PKB–transfected cells, the okadaic acid–induced Ser473 phosphorylation was at the same level as the Ser473 phosphorylation induced by PDGF (Figure S2A, densitometry). However, in the case of phospho-Thr308, the okadaic acid signal was reduced compared to phosphorylation triggered by PDGF stimulation (Figure S2A, densitometry). The reduced phosphorylation of Thr308 led to the hypothesis that Thr308 was inaccessible in the cytoplasmic PDK1 complex due to PH domain steric hindrance. If this hypothesis is correct, then the removal of the PH domain should alter the kinetics of Thr308 phosphorylation in response to okadaic acid. Thr308 phosphorylation of the deletion mutant RFP-ΔPH PKB was more extensive than the wild-type PKB (Figure S2C). This provided evidence that, in the cytoplasm, Thr308 was not fully accessible to PDK1 and hence could not be phosphorylated.

To verify that in the RFP-ΔPH-PKB mutant Thr308 phosphorylation was due to PDK1, NIH3T3 cells were cotransfected with RFP-ΔPH PKB and GFP-PDK1 wild-type or the PH domain mutant GFP-PDK1 RRR (Figure S2D). In all cases, a prominent phosphorylation of Thr308 independent of PDGF was detected. Therefore, it is established that when the PKB PH domain is absent, Thr308 could be phosphorylated by PDK1 within the cytosolic complex. Thus, for maintenance of PKB in a Thr308-dephosphorylated state under basal conditions, its PH and kinase domains are predicted to interact to obscure Thr308 accessibility.

### PKB PH and Catalytic Domains Interact in the PKB Inactive State

**Figure 2:**
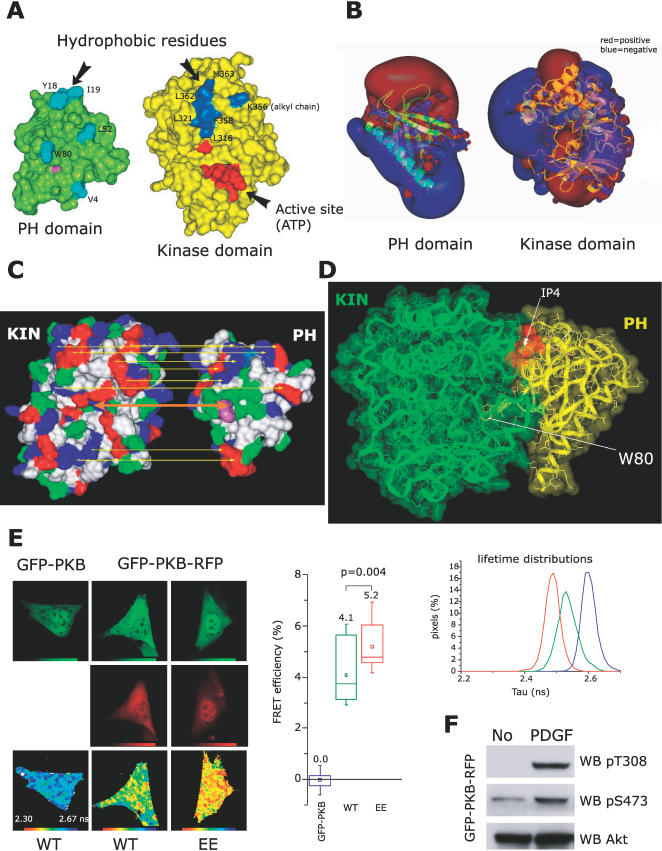
PKB PH and Catalytic Domains Interact in the PKB Inactive State (A) The solvent-accessible surface of the domains are shown in green (PH) and in yellow (kinase) and the hydrophobic patches are indicated. The IP_4_ binding site is in magenta (located to the rear of PH domain) and the ATP active site is in red. (B) The electrostatic potential maps of PKB PH and kinase domains are calculated in the complexed form, based on crystal structures of PKBα PH domain. A strong complementarity between the positive and negative lobes of the PH and kinase domains is seen. (C) Acidic (red), basic (blue), and polar (green) residues are presented here. The Trp80 is shown in purple and Arg25 is shown in light blue. The yellow arrows show the complementarity between the acidic and basic residues in each domain. The orange arrow shows the docking site of Trp80 in the kinase domain cleft. (D) The model of complexed PKBα PH domain (yellow) and the rebuilt catalytic domain model (green) is based on the hydrophobicity in an aqueous environment. To visualise the binding site of the lipid head group in the complex, IP_4_ is represented as CPK (Corey-Pauling-Koltun) orange spheres. Note that IP_4_ cannot be positioned unless the PH and kinase domains are separated. (E) NIH3T3 cells were transfected either with GFP-PKB or GFP-PKB-RFP (WT) or mutant (EE). The lifetimes are in pseudo-colour scale ranging from 2.30 to 2.67 ns. The graph shows the lifetime distributions: GFP-PKB (blue), GFP-PKB-RFP WT (green), and EE (red). The statistical analysis shows the highly significant variation of FRET efficiency between WT and EE (*p* = 0.004). (F) Expression of GFP-PKB-RFP and phosphorylation on Thr308 and Ser473 sites is determined by Western blot using total protein (WB Akt) or phosphospecific antibodies as indicated.

To test the above hypothesis and determine the possible interactive sites of the PH and kinase domains, molecular modeling was exploited. The calculations of lipophilicity potentials of the PH and kinase domains resulted in finding two isolated hydrophobic patches that were located on the kinase domain (Figure 2A, in blue). On the PH domain, four small hydrophobic patches were found (Figure 2A, in cyan) and were located on the same side of the protein. To test the validity of this molecular model, the electrostatic potential maps of each domain were calculated. The complementarity observed between the positive (red) and negative (blue) lobes for the two domains (Figure 2B) was almost perfect. The upper positive lobe of the PH domain corresponded to the upper negative lobe of the kinase domain, and reciprocally, the negative lobe on the PH domain corresponded to a positive lobe on the kinase domain. Figure 2C shows the complementarity between the acidic and the basic residues in the kinase and PH domains of PKB. The yellow arrows indicate the pair of complementary residues on each domain. The fit of the hydrophobic interactions predicts that the Trp80 on the PH domain, at the extremity of the variable loop 3, inserts inside a deep cleft in the kinase domain around residues Lys297, Glu298, and Glu314 (Figure 2D). Furthermore, the crystal structure of the isolated PKB PH domain showed that Trp80 may have an important role in the interaction of the two domains of PKB [7].

From the complementarity of the basic and acidic residues located around Trp80 in the PH and kinase domains, it was envisaged that mutations of these regions would disrupt the docking of these domains. The region where Trp80 interacts in the kinase domain is indicated by an orange arrow (Figure 2C). The two polar residues Gln79 and Thr82, surrounding Trp80, were mutated to Glu. These mutations were predicted to result in disruption of the two domains since the acidic residues repulse one another instead of the polar residues of the PH domain interacting with acidic residues of the kinase domain.

To monitor and validate in situ our hypothesis on the docking sites of PKB-PH and kinase domains and specifically the Gln79Glu/Thr82Glu mutant (PKB-EE mutant), a PKB reporter was constructed. Full-length PKBα with EGFP on the N terminus and monomeric red fluorescent protein (mRFP) on the C terminus (GFP-PKB-RFP) was genetically encoded. Variations in PKB conformation were monitored by changes in FRET efficiency. NIH3T3 cells were transfected with PKB tagged only with GFP to use as the reference lifetime (GFP-PKB) or with the double-tagged GFP-PKB-RFP reporter (Figure 2E). Under basal conditions, a change in GFP lifetime and FRET efficiency in the GFP-PKB-RFP reporter construct was monitored (Figure 2E, box and whiskers plots). The dynamics of the double-tagged reporter is shown in Figure S5. It is of note that double-tagged PKB can be phosphorylated on both Thr308 and Ser473 (Figure 2F). The FRET signal under basal conditions was indicative of the close proximity of the two domains in the inactive conformer of PKB. Upon the expression of the Gln79Glu/Thr82Glu mutant (GFP-PKB EE-RFP), a further decrease in the lifetime of the reporter (red lifetime distribution curve) was detected and the FRET efficiency significantly increased to 5.2 (Figure 2E, box and whiskers plots). As predicted from molecular model, the double-Glu mutation perturbed the juxtaposition of the PH and kinase domains as reflected in this altered N-terminal to C-terminal FRET (Figure 2E).

### Phosphoinositide-Dependent PKB Conformation Change at the Plasma Membrane

The molecular model implied that a close interaction between the PH and kinase domains was formed as predicted from the complementary surfaces and shown by the extremely significant variations in FRET efficiency between wild-type and mutant PKB. This inactive conformer will be referred to as the “PH-in” conformation. In order for PDK1 to access Thr308, it was predicted that the PH domain alters its association with the kinase domain on binding to lipids, i.e., a “PH-out” conformation.

**Figure 3:**
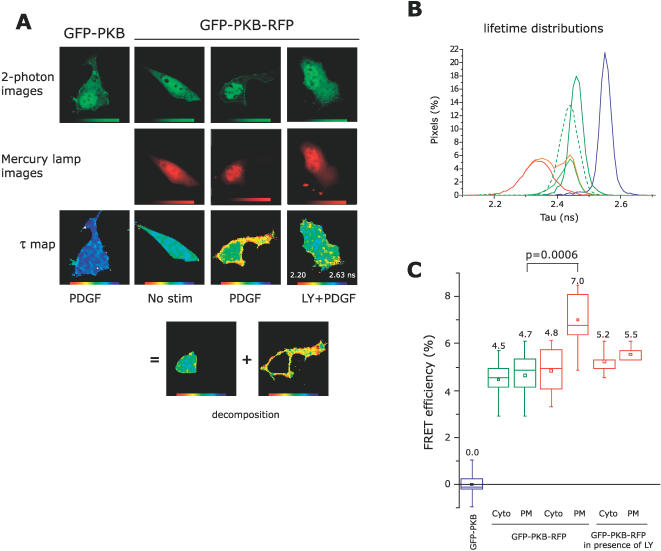
Phosphoinositide-Dependent Change in PKB Conformation at the Plasma Membrane (A) NIH3T3 cells were transfected either with GFP-PKB or GFP-PKB-RFP. Upon PDGF treatment, GFP-PKB-RFP translocated to the plasma membrane and changed in conformation. The lifetimes are represented in pseudo-colour scale ranging from 2.20 to 2.63 ns. As indicated, the cells were pretreated for 20 min with LY294002 before PDGF stimulation. LY294002 prevented the translocation of GFP-PKB-RFP to the plasma membrane and its change in conformation but did not affect the FRET efficiency in the cytoplasm. (B) The lifetime distributions are shown on the graphs: GFP-PKB control (blue), GFP-PKB-RFP (green), and GFP-PKB-RFP upon PDGF (orange) and treated with LY294002 before PDGF stimulation (dashed green). The separation of the lifetime pixels at the plasma membrane (red) and cytoplasm (light green) is shown for GFP-PKB-RFP upon PDGF stimulation. (C) The statistical analysis presented as box and whiskers plots shows the extremely significant change in FRET efficiency (*p* = 0.0006) of GFP-PKB-RFP before and upon PDGF stimulation at the plasma membrane (PM) and the loss of FRET change when pretreated with LY294002.

To test this hypothesis, the GFP-PKB-RFP reporter was expressed in NIH3T3 cells and its behaviour was monitored upon stimulation with PDGF. Figure 3A illustrates that PDGF induced a change in the conformation of PKB concomitant with its translocation to the plasma membrane. The lifetime distribution curves demonstrated the changes in lifetime (Figure 3B), as did the FRET efficiencies (Figure 3C). By separating the plasma membrane pixels from those of the cytoplasm, the mean FRET efficiency of each compartment was calculated to be 7.0 and 4.8, respectively (Figure 3C, *p* = 0.0006). The cytoplasmic FRET efficiency in PDGF-treated cells in this acute response was similar to basal level FRET efficiency (Figure 3C), indicating that the “PH-in” conformer, unless recruited to the plasma membrane, remained unchanged. Pretreatment with LY294002 inhibited the conformational change in wild-type PKB as seen by the overlap of the distribution curves (Figure 3B, green and dashed green lines). Similarly, the nonbinding PH domain mutant of PKB (RFP-PKB R25C-RFP) was not recruited to the plasma membrane and did not change conformation upon stimulation with PDGF (unpublished data). In conclusion, the association of the PH domain with phosphoinositides at the plasma membrane induced a change in PKB conformation by stabilising the “PH-out” conformer and so permitting Thr308 phosphorylation.

### PKB Conformation Dynamics Revealed in Live Cells

**Figure 4:**
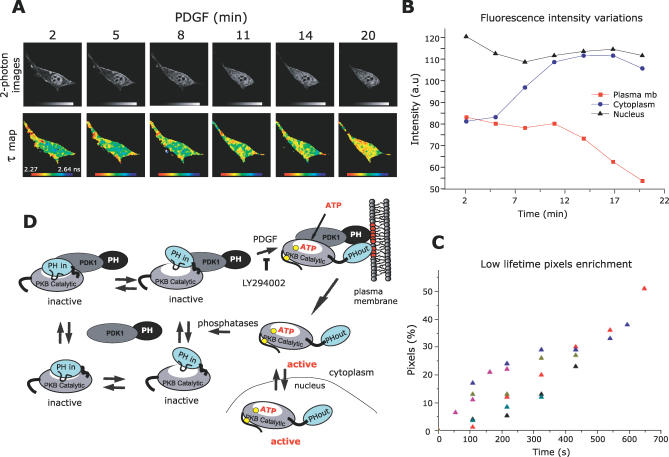
PKB Conformation Dynamics Revealed in Live Cells (A) A series of two-photon FLIM images of a GFP-PKB-RFP–transfected NIH3T3 cell were acquired over a period of 20 min (every 54 s). The top panels represent intensity images of the donor GFP chromophore in the transfected cell after two photon excitation. The bottom panels represent the calculated lifetime maps of GFP-PKB-RFP over time. After treatment with 60 ng/ml PDGF, cells were observed under the microscope. Acquisition took place once the construct had translocated to the membrane. The lifetimes are represented in pseudo-colour scale ranging from 2.27 to 2.64 ns. (B) The variations of fluorescence intensity at the plasma membrane (red squares) in the cytoplasm (blue circles) and in the nucleus (black triangles) were detected over time. (C) The time-course graph presents the enrichment in short lifetime pixels of six different experiments. Each coloured triangle presents a cell. The enrichment of short lifetime pixels is calculated as described in Supporting Information. (D) Under basal conditions, PKB and PDK1 form a complex in the cytoplasm where the associated and dissociated forms are in dynamic equilibrium. Four possible equilibrium states of PKB–PDK1 complex in its inactive conformation are represented. In the PKB inactive conformation, PKB PH and kinase domains interact, noted as “PH-in.” The equilibrium between a “tighter” and a “looser” interaction of PKB PH with its kinase domain is also represented. Upon PDGF stimulation, the PKB–PDK1 complex is recruited to the plasma membrane due to interaction with phosphoinositides (in red). The equilibrium of PKB–PDK1 interaction is shifted toward the associated form, a process compromised by LY294002. The interaction of PKB PH domain with the lipids induces a change in conformation of PKB, noted as “PH-out.” In this conformation, Thr308 becomes accessible to PDK1 (the phosphorylation sites are represented by yellow circles). After Ser473 phosphorylation and loading of ATP, PKB dissociates from the plasma membrane in its active conformation to return to the cytoplasm and the nucleus. PKB re-adopts an inactive conformation upon dephosphorylation of its Thr308 and Ser473 sites.

It has been suggested that PKB in its active state dissociates from the membrane and can phosphorylate soluble targets [22]. However, the “PH-in” conformer may be obscuring its substrate-binding pocket so that PKB's downstream targets will not have access to it. Thus, we speculated that upon dissociation from the plasma membrane, phosphorylated PKB would remain in a “PH-out” conformer where it can phosphorylate cytosolic substrates. To follow the dynamics of the “PH-out” conformer on dissociation from the plasma membrane, the behaviour of the PKB conformation reporter (GFP-PKB-RFP) was assessed in live cells (Figure 4). Cells were transfected with the reporter, and upon stimulation with PDGF, images were acquired every 54 s for up to 20 min (Figure 4A). The intensity images and the quantification of fluorescence intensity illustrated a replenishment of the cytoplasmic fluorescence, which coincided with a decrease in the fluorescence intensity at the plasma membrane (Figure 4B) upon recruitment of PKB. The lifetime maps show that the translocated PKB changed conformation, and over time, the populations of the shorter lifetime pixels were augmented in the cytoplasm and the nucleus (Figure 4A). The quantification of these lifetime variations in six different experiments demonstrated that the mean FRET efficiency, upon stimulation, increased gradually over time (Figure 4C). This indicated that during the 20 min of filming, the de novo PDGF-activated “PH-out” PKB conformer dissociated from the membrane and became distributed in the cytoplasm and the nucleus.

## Discussion

Assembling these observations produces a new molecular model for PKB activation dynamics (Figure 4D). Prior to stimulation, PKB and PDK1 form a complex in the cytoplasm that is in constant equilibrium between the associated and dissociated forms. Under basal conditions, PKB is maintained in its inactive form by the interaction of its PH and kinase domains (“PH-in”). The “PH-in” conformation is responsible for preventing the phosphorylation of Thr308 by the associated PDK1. The cytoplasmic and membrane interactions of PKB and PDK1 are similar in nature. These proteins are in dynamic equilibrium, and it is their corecruitment that concentrates them at the plasma membrane to shift the equilibrium state and form more complex at this location. PKB PH domain interaction with phosphoinositides and its concomitant change in conformation (“PH-out”) form the critical step that allows the associated, corecruited PDK1 to phosphorylate Thr308.

In live cells, the dynamics of PKB activation shows that it remains in the “PH-out” conformer upon dissociation from the plasma membrane. Active PKB accumulates in both the cytoplasm and the nucleus. This observation suggests that the phosphorylated kinase domain has a lower affinity for the PH domain, thereby sustaining the “PH-out” conformer, whereas the dephosphorylated kinase domain has a higher affinity for the PH domain. This higher affinity for the PH domain, in the inactive form of PKB, maintains the “PH-in” conformer. The “PH-out” conformer probably sustains its activity until phosphatases render it inactive; once inactivated by dephosphorylation, the “PH-out” conformer returns to the “PH-in” conformation. Aside from providing a rationale for observations related to the mechanism of PKB activation, this detailed model makes interesting predictions regarding the potential sites for influencing PKB and its activation. Specifically, it is predicted that compounds influencing the “PH-in” versus “PH-out” conformers will influence the activation state of PKB. This may have implications both in cancer where PKB is aberrantly activated and in diabetes where transient PKB activation might be insulinomimetic.

## Materials and Methods

### Materials and antibodies.

Mowiol 4–88, LY294002 in solution, okadaic acid, and sodium salt were from Calbiochem (Merck KGaA, http://www.merck.de). Human PDGF was from R&D Systems (http://www.rndsystems.com). Cyan 3 dye was from Amersham Biosciences UK (http://www.amersham.com). Phospho-Akt Thr308 and Ser473 as well as pan Akt were from Cell Signaling (New England BioLabs UK, http://www.neb.com). Anti-HA and -GFP antibodies were in-house monoclonal antibodies. Sodium borohydride (NaBH_4_) and other chemicals were from Sigma-Aldrich Company Ltd. (http://www.sigmaaldrich.com). Poly-l-lysine–coated glass coverslips were from Marathon Laboratory Supplies (London, United Kingdom).

### Fusion constructs design and direct site mutagenesis.

pRSET B-mRFP was kindly provided by Dr Roger Tsien (University of California). The construct pCMV5-EGFP-PDK1 (human PDK1) was described previously [23].

To create mRFP-PKB, mRFP was PCR amplified from pRSET B-mRFP with the following oligos: mRFP sense: agagaattcgccgccaccatggcctcctccgaggacgtcatcaaggagttcatgcgc, and mRFP antisense: atcgaattctgcaccggtggagtggcggccctcggcgcgctcgtactgttcc. PCR-amplified mRFP was then subcloned into the murine AKT1 (described elsewhere [24]) in an EcoRI site in N terminus of HA-PKB. An extra EcoRI site (in the vector) C terminus of PKB was eliminated by mutagenesis prior to the subcloning of RFP using the following primers: EcoRI less-sense: gccacgctgtcctccgcaattcgagctctagaggatcccgg, and EcoRI less-antisense: ccgggatcctctagagctc gaattgcggag gacagcgtggc.

mRFP-ΔPH-PKB was made by PCR-amplifying mRFP and put in instead of EGFP using the flanking restriction sites NheI-BglII in pEGFP-ΔPH-PKB vector (previously described [25]). The oligo used for the PCR amplification of mRFP, kindly provided by Dr Johanna Durgan (CRUK, London, England), are NheI sense: aatatagctagcatggcctcctccgaggacg, and BglII antisense: atataaagatcttgcgccggtggagtggcggccctc.

The different mutants of PKB and PDK1 were obtained by direct site mutagenesis using the Quick Change mutagenesis kit from Stratagene (Stratagene Europe, http://www.stratagene.com). The oligonucleotides sequence used for each mutant is as follows: PKB-R25C sense: caagacctggcggccatgctacttcctcctcaagaatgatggcacc, PKB-R25C antisense: ggtgccatcattcttgaggaggaagtagcatggccgccaggtcttg, PKB-S473A sense: cgcaggccccacttcccccagttcgcctactcggccagcagcacg, PKB-S473A antisense: cgtgctgctggccgagtaggcgaactgggggaagtggggcctgcg, PKB-T308A sense: gacggtgccaccatgaaggccttttgcggcacacctgagtacctg, PKB-T308A antisense: caggtactcaggtgtgccgcaaaaggccttcatggtggcaccgtc, PDK1-RRR-LLL sense: gcggaagggtttatttgcactactactacagctgttgctcacagaagg, PDK1-RRR-LLL antisense: ccttctgtgagcaacagctgtagtagtagtgcaaataaacccttccgc, PKB-EE sense: cctttatcatccgctgcctggagtggaccgaagtcattgagcgcaccttc, PKB-EE antisense: gaaggtgcgctcaatgacttcggtccactcc aggcagcggatgataaagg, PDK1-L155E sense: catttcaggacgacgagaaggagtatttcggccttagttatgcc, PDK1-L155E antisense: ggcataactaaggccgaaatactccttctcgtcgtcctgaaatg, PDK1-R131A sense: ggtcccctatgtaaccagagaggcggatgtcatgtcgcgcctgg, and PDK1-R131A antisense: ccaggcgcgacatgacatccgcctctctggttacataggggacc.

pEGFP-HA-Akt construct was described previously (murine AKT1 [14]; to simplify, we called this construct GFP-PKB).

GFP-PKB-RFP was created by putting PCR-amplified mRFP (EcoRI-XbaI) instead of YFP in the vector pCDNA3-GFP-PKB-YFP (described previously [26]), using the following oligos: mRFP-Ct sense: agagaattcgcctcctccgaggacgtcatcaaggagttcatgcgcttcaagg, and mRFP-Ct antisense: atctctagattatgcaccggtggagtggcggccctcggcgcgctcg tactgttcc.

Note that an extra XbaI site had to be removed by QuickChange in the pCDNA3-GFP-PKB-YFP construct before the subcloning of mRFP (EcoRI-XbaI).

### Cell culture and transfections.

NIH3T3 cells from ATCC (http://www.atcc.com) were maintained in DMEM 10% donor calf serum and seeded at 150,000 in a well of a six-well plate or in a MatTek dish. The transfection was done with 2 μg of DNA of the different constructs (2 + 2 μg for cotransfections) using LipofectAMINE/PLUS reagent (GIBCO BRL, http://www.invitrogen.com) in OptiMEM medium (GIBCO BRL) as recommended by the manufacturer. The cells were left for 3 h in the transfection mix and then the medium was removed and replaced with DMEM 10% donor calf serum. The experiments were performed 24 or 48 h after transfection.

### Lysis conditions and Western blot analysis.

After stimulation or treatment as indicated, the cells were lysed for 15 min on ice in lysis buffer (20 mM Tris-HCl [pH 7.4], 150 mM NaCl, 100 mM NaF, 10 mM Na_4_P_2_O_7_, and 10 mM EDTA supplemented with Complete protease inhibitor cocktail tablet [Roche, http://www.roche.com)]. To terminate the reaction, 4× SDS sample buffer (125 mM Tris-HCl [pH 6.8], 6% SDS, 20% glycerol, 0.02% bromophenol blue supplemented with 10% β-mercaptoethanol) was added, and the samples were boiled for 5 min. The proteins were separated on a 10% SDS-PAGE gel. The gels were then transferred onto PVDF membrane (Immobilon P; Millipore, http://www.millipore.com), incubated in blocking buffer TBS-T (10 mM Tris-HCl [pH 7.4], 150 mM NaCl, 0.05% Tween-20) supplemented with 3% BSA for 1 h, washed in TBS-T/1% milk for 1 h, and incubated with the different antibodies phospho-Akt (Thr308) antibody (rabbit polyclonal from Cell Signaling, http://www.cellsignal.com) or phospho-Akt (Ser473) (rabbit polyclonal antibody raised against the C-terminus phosphopeptide HFPQF*p*SYSASS of Akt1) at 1:1,000 for 2 to 3 h in TBS-T/3% BSA. Anti-GFP (mouse monoclonal antibody, CR-UK) was used at 1:5,000 for 1 h. The secondary HRP-antibodies were used at 1:5,000 in TBS-0.2% Tween-20 in 5% milk for 1 h. Western blots were revealed by incubation with ECL (Amersham Biosciences). Density analysis of bands was done with NIH ImageJ 1.33u (National Institutes of Health, http://rsb.info.nih.gov/ij). The analysis was performed by substracting the background of the autoradiography. The intensity of the bands was normalised to the total amount of protein, and the relative variations were plotted.

### FRET by two-photon FLIM.

NIH3T3 cells were seeded at 150,000 on 30-mm glass-bottom tissue culture dishes (MatTek) and transfected as described above. At 24 h after transfection, the cells were treated in DMEM containing 10% donor calf serum. The cells were washed twice with PBS and then fixed in 4% paraformaldehyde in PBS for 10 min. The dishes were washed twice with PBS and then 2 ml of PBS supplemented with 2.5% (w/v) 1,4-diazabicyclo-[2.2.2]-octane (DABCO) as an antifade was added to the dishes. The images were acquired straightaway or stored at 4 °C.

For live experiments, the cells were seeded and transfected as described above. They were treated with 60 ng/ml PDGF in the same medium, and the lifetime was measured every 54 s with two-photon FLIM for 20 min. The live experiments were repeated six times to verify reproducibility.

Details about the method to detect FRET by time domain FLIM can be found elsewhere [27]. All the images were acquired on a modified TE 2000-E inverted microscope (Nikon Ltd., http://www.nikon.co.uk). The fluorescence lifetime measurements were obtained using an SPC 830 time-correlated single photon counting (TCSPC) electronic card (Becker and Hickl, http://www.Becker-Hickl.de) in the reverse stop-start mode. A mode-locked tuneable Ti:sapphire laser (Mira 900; Coherent, http://www.coherentinc.com) pumped by a solid-state diode laser (Verdi; Coherent) was used. For two-photon excitation of EGFP, the laser was tuned at 890 nm and pumped at 6 W. The Ti:sapphire laser generates 125-fs pulses with a repetition rate of 76.26 MHz and an average power output of 450 mW. The laser beam was focused with a ×40 oil immersion objective lens (1.3 NA, Plan-Fluor; Nikon Ltd.). The fluorescence was detected through the same objective in a descanned configuration and detected with a fast photomultiplier (Hamamatsu 7400, Hamamatsu, http://www.hamamatsu.com) after filtering with a bandpass filter (510 ± 10 nm, Chroma Technology Corp, http://www.chroma.com).

### Acquisition parameters.

The acquisition parameters were adjusted to avoid photobleaching [28] and to detect enough photons such that the signal-to-noise ratio was high enough for data analysis. The power of the laser beam was decreased to 9 mW with a neutral optical density filter. The acquisition time was maintained at 300 s for less-intense cells but could be reduced to 30 s for brighter ones. A dwelling time of 17 μs per pixel with a resolution of 256 × 256 pixels was used. Photobleaching was evaluated by comparing the photon count upon time, maintaining the photobleached fraction of EGFP inferior to 2% in all the acquisitions. Depending on the fluorescence intensity of the cells, the maximum photon count of the intensity decay was obtained from 100 to 1,000 counts after a spatial binning of 11 × 11 pixels. The binning was maintained constant throughout all experiments. The count rate frequency was below 10^5^ photons/s to avoid any pulse pile-up. Epifluorescence intensity images of both EGFP and mRFP were acquired with the mercury lamp source of the TE 2000-E microscope and the fluorescence detected by a cooled CCD camera (Hamamatsu ORCA-ER). The cubes set in the TE 2000-E microscope turret were FITC (Nikon Ltd.) for EGFP and G-2A (Nikon Ltd.) for mRFP. No bleed-through was detected.

### Data analysis.

The analyses were performed using in-house software developed by the Advanced Technology Development Group at the Gray Cancer Institute.

FRET measurements were based on the reduction of the fluorescence lifetime of the donor upon specific quenching with the acceptor. The fluorescence lifetime of the donor (τ_D_) was obtained by fitting the intensity decay with a monoexponential fit. When cells were transfected with both EGFP-PDK1 and mRFP-PKB, two distinct populations can be considered in first approximation. The first one consists of the noninteracting EGFP-PDK1, with a fluorescence lifetime τ_D_. The second consists of the interacting EGFP-PDK1 with mRFP-PKB. The formation of the complex EGFP-PDK1/mRFP-PKB leads to a transfer of energy from the donor to the acceptor, inducing a decrease in the fluorescence lifetime of EGFP-PDK1. When measuring the fluorescence intensity decay of EGFP-PDK1 within the cell, photons from both interacting and noninteracting EGFP-PDK1 were collected. Similarly, when transfected with EGFP-PKB-mRFP, the conformational change of the fusion protein leads to a variation of the FRET efficiency due to a variation of distance between the two fluorescent proteins. In both types of experiments, the intensity decays should follow a multiexponential law. However, biexponential fits necessitate a high signal-to-noise ratio. A minimum photon count of 1,000 at the maximum of the intensity decay is required. Considering the limitations inherent to experiments in cells, such as photobleaching and low concentration of EGFP, the number of photons was insufficient to accurately proceed with a multiexponential analysis. Moreover, to accurately identify the contribution of two lifetimes, it is preferable that they differ by at least a factor of 2, which is generally not the case with GFP-like fluorescent proteins. Therefore, to detect FRET, we measured the relative variation of lifetime obtained from monoexponential fit [29]. In each experimental condition, a minimum of ten cells were acquired and analysed. The experiments were repeated at least three times for reproducibility. The intensity images were converted to fluorescence lifetime maps by fitting the fluorescence intensity decay at each pixel. The fluorescence lifetime was represented in pseudo-colour with the same scale so that colours were comparable in each experiment. Fluorescence lifetime distributions were represented by histograms. The histograms were normalised to the total number of pixels of the cell.

### Statistical analysis.

We used a nonparametric Mann-Whitney test to compare the medians of the two data sets for the two-photon FLIM data using GraphPad InStat software (version 3.0 for Mac-2001; http://www.graphpad.com). To interpret the distribution of data, box and whiskers plots were used. The box and whiskers plot is a histogram-like method for displaying upper and lower quartiles, and maximum and minimum values in addition to median [30].

An unpaired *t*-test with Welch correction was used for frequency FLIM data to determine the significance (*p*-values at 95% confidence interval) of variation in lifetime of GFP-PDK1 in the presence or not of HA-PKB (Cyan 3 dye [Cy3]-HA antibody). Fifty-six or more cells were analysed per experiment; the number of experiments were at least ten.

### Live imaging analysis.

Enrichment in short lifetime pixels over time was quantified from the lifetime histograms. The lifetime histogram for each time point was normalised by the total number of pixels *F_i_ = f_i_/N_i_*, where *f_i_* and *N_i_* are, respectively, the lifetime histogram and the total number of pixels at time point *t = i*. The normalised histogram was subtracted from one of the first images *ΔF_i_ = F_I_* − *F_0_*, where *F_i_* and *F_0_* are, respectively, the normalised histograms at *t = i* and *t = 0*. The difference was integrated over *τ* and the enrichment in short lifetime pixels obtained by taking its maximum *η_i_ = Max ∫ΔF_i_dτ*. The enrichment *η_i_* was plotted as a function of time. Cells were selected when translocation could be observed. Lifetime measurements were acquired every 54 s. The enrichment in short lifetime pixels was calculated as described above as a function of time. Some cells presented transient periods during which no significant lifetime variation was measurable. For those cells, the enrichment in short lifetime pixels was represented only after this transition period.

### FRET by frequency domain FLIM.

NIH3T3 cells were seeded at 30,000 on poly-l-lysine–coated glass coverslips in 24-well plates and transfected as described above but with a maximum of 0.4 μg of DNA. After treatment, the cells were washed twice in PBS and fixed in 4% paraformaldehyde (PFA) in PBS for 10 min. The cells were washed in PBS and permeabilised with 0.2% Triton X-100 in PBS for 5 min before being incubated with 1 mg/ml NaBH_4_ in PBS for 5 min. Following another wash with PBS and 10-min incubation in the blocking buffer PBS/1% BSA, the cells were incubated for 1 h with 0.22-μm filtered anti-HA antibody labeled with Cy3 in the same buffer. The coverslips were washed twice with PBS and once with water and mounted on slides with the mounting medium Mowiol 4–88 containing 2.5% (w/v) DABCO as an antifade. The slides were then analysed in the frequency domain FLIM.

A detailed description of the FRET monitored by frequency domain FLIM can be found elsewhere [31]. We have monitored lifetime detection in the frequency (phase) domain. Phase methods provide an average lifetime where sinusoidally modulated light is used to excite the sample. The lag in the emitted fluorescence signal permits measurement of phase (τ_p_) and modulation depth (τ_m_) of the fluorescence. The lifetime, <τ>, is the average of phase shift and relative modulation depth (τ_m_ + τ_p_)/2 of the emitted fluorescence signal. We monitor the decrease in lifetime of donor (EGFP) in presence of the acceptor (mRFP or Cy3 dye).

All images were taken using a Zeiss Plan-APOCHROMAT ×100/1.4 numerical aperture, phase 3 oil objective, with images recorded at a modulation frequency of 80.218 MHz. GFP-PDK1 was excited using the 488-nm line of an argon/krypton laser, and the resultant fluorescence was separated using a combination of dichroic beamsplitter (Q505 LP; Chroma Technology Corp.) and narrow band emission filter (BP 514/10; Lys & Optik, Lyngby, Denmark).

### Molecular modeling: Sequence homology building.

**Figure 5:**
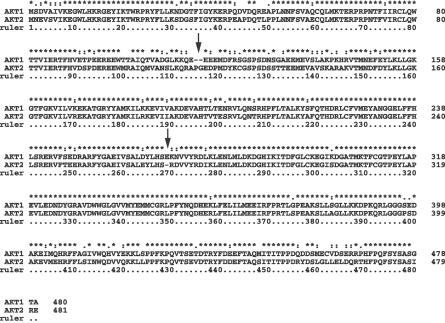
Sequence Alignment between Akt-1 and Akt-2 Kinase Domain

For the PH domain, we used the x-ray structure with Protein Data Bank (http://www.rcsb.org/pdb) code 1UNP [7]. In this structure, only the two first amino acids were lacking and were not rebuilt (Figure 5). There is no available structure for the kinase domain of human Akt-1 (PKBα), but there are several structures for the homolog human Akt-2 kinase domain (PKBβ). Among them, one of the most complete is 1GZN with a resolution of 2.5 Å [11]. In this side chain, the following amino acids are lacking: 146–147, 198–206, 253, 296, and 442. These were rebuilt prior to any experiment using the Homology module of Insight II (Accelrys, http://www.accelrys.com). Because the two proteins (Akt-1 and Akt-2) have a very high homology rate (98.8% with 82.3% of strict identity), building a model from this homology sequence was straightforward. Moreover, in the chosen alignment (Figure 5), there is a difference of only one amino acid between the two sequences that results from a two–amino acid deletion after position 114 and a one–amino acid insertion at position 268 (Figure 5, arrows). Alignment of sequences was performed using the Homology multiple-sequence aligner software via the PAM (120 or 250) matrices or the ClustalW software (http://www.ebi.ac.uk/clustalw) as implemented within Homology module. In these latter cases, alignments were performed using the Blosum and Gonnet matrices. The crude model of each domain, after full protonation at pH 7.4, was refined by several cycles of splice repairs on the loops and relaxation on the SCR's side by 100 steps of Steepest Descent and 500 cycles of conjugate gradient. Small 5-ps molecular dynamics runs were performed on each loop in order to relax the strains. Finally, the entire backbone was fixed and the side chains were fully minimised with conjugate gradient method until a root-mean-square of 0.1 kcal/Å/mol was obtained. The minimisation process was resumed by applying a tethering constraint to the backbone. This constraint was slowly lowered (starting with a 100 kcal/Å/mol) force constant) in a stepwise manner so that at each step the energy of the root-mean-square gradient was less than 0.1 0.1 kcal/Å/mol (steps are 100, 50, 25, 10, and 0 0.1 kcal/Å/mol).

### Molecular modeling: Potential maps calculations.

Lipophilicity potentials were calculated using a previously described software [32] that calculated on each node of a 1-Å cubic grid the sum of the lipophilicity contribution of each nonhydrogen atom as a function of distance. On the kinase domain, a unique patch of hydrophobic potential is located on the same side as the ATP active site with the following amino acids: Leu316, Leu321, Lys356 (alkyl chain only), Phe358, Leu362, and Met363. For the PH domain, four small hydrophobic patches are also located on the same side of the protein and on a “plateau” with the following amino acids: Val4, Tyr18, Ile19, Leu52, and Trp80.

The Molecular Electrostatic Potential (MEP) maps were drawn within the software PYMOL (version 0.97) after calculations performed using the implemented APBS plug-in. This last package enabled us to efficiently evaluate electrostatic properties by solving the Poisson-Boltzmann equation [33].

All possible hydrophobic and electrostatic interactions were properly fitted. The Trp80 at the extremity of a loop inserts precisely inside a deep cleft in the kinase domain with Lys297, Glu298, and Glu314 located around it. Moreover, in this configuration, the C terminus of the PH domain is on the same side as the N terminus of the kinase domain. In this model, the PH domain completely blocks the entry of the ATP active site and Thr308 will not be accessible.

## Supporting Information

Figure S1 PKB–PDK1 Interaction Monitored by Frequency Domain FLIM (A) Fluorescence frequency domain FLIM images of NIH3T3 cells transfected with GFP-PDK1 alone or GFP-PDK1 together with HA-PKB as indicated. The top panels represent intensity images of the donor fusion construct GFP-PDK1. The middle panels show of the use of an epifluorescence OCRA camera in addition to frequency FLIM images to provide a better quality image of the FRET partners (donor in green and acceptor in yellow). The bottom panels represent the calculated average lifetime maps of GFP-PDK1 alone or in presence of the acceptor HA-PKB recognized with Cy3-labeled anti-HA antibody after fixation. The lifetimes are shown in a pseudo-colour scale ranging from 1.7 to 2.1 ns.
(B) The two-dimensional diagram presents the statistical analysis of the lifetimes phase (τ_p_) and modulation (τ_m_) of cells taken from at least ten different experiments (*n* being the total number of cells acquired). The diagonal shift toward the lower lifetimes indicates the occurrence of FRET. The conditions are GFP-PDK1 alone (black circle), GFP-PDK1 in presence of PDGF (black square), GFP-PDK1 with Cy3-HA-PKB (white circle), and GFP-PDK1 with Cy3-HA-PKB upon 5-min PDGF treatment (red circle). For the statistics, an unpaired *t*-test with Welsh correction was used. An extremely significant *p*-value (*p* < 0.0001) was obtained at 95% confidence interval by comparing nonstimulated GFP-PDK1 + Cy3-HA-PKB cells (white circle) with GFP-PDK1 cells stimulated with PDGF (black square). An extremely significant *p-*value (*p* < 0.0001) was also obtained when comparing PDGF-stimulated GFP-PDK1 cells cotransfected with Cy3-HA-PKB (red circle) versus nonstimulated cells (white circle). The difference between GFP-PDK1 nonstimulated cells (black circle) and PDGF-stimulated cells (black square) was not significant (*p* = 0.10).
(9.9 MB EPS)

Figure S2 Regulation of PKB Phosphorylation in the Cytoplasm by Its PH Domain and Phosphatases (A) NIH3T3 cells expressing RFP-PKB were stimulated with PDGF as indicated or treated for increasing periods of time with 1 μM okadaic acid alone. The phosphorylation of RFP-PKB on Thr308 and Ser473 was determined with phosphospecific antibodies, and the expression by Western blot with an anti-PKB antibody (pan Akt). The lower panel represents Thr308 phosphorylation of endogenous PKB in the same experiment. The quantification of the bands was performed by densitometry. Ser473 is shown in black bars, and Thr308 is shown in gray bars. The phospho bands were normalised to total protein.
(B) The same experiment as in (A) was performed but with NIH3T3 cells expressing RFP-PKB R25C.
(C) The same experiment as in (A) was performed but with NIH3T3 cells expressing RFP-ΔPH PKB. Similar levels of Thr308 phosphorylation of endogenous PKB in (A), (B), and (C) allow a direct comparison between the level of Thr308 phosphorylation of exogenous RFP-PKB or mutants shown in the middle panels of (A), (B), and (C).
(D) NIH3T3 cells were transfected with RFP-PKB or RFP-ΔPH PKB alone or together with GFP-PDK1 (WT) or PH domain defective GFP-PDK1 RRR. The phosphorylation of RFP-PKB and RFP-ΔPH PKB on Thr308 was determined with a phosphospecific antibody, and the expression by Western blotting with an anti-PKB antibody (pan Akt). Levels of expression of GFP-PDK1 and GFP-PDK1 RRR in the bottom panel were determined with an anti-GFP antibody.
(E) NIH3T3 cells expressing RFP-PKB were pretreated for 20 min with LY294002 as indicated and stimulated with PDGF for 5 min. The expression of RFP-PKB was determined by Western blot with an anti-HA antibody, and the phosphorylation of Thr308 and Ser473 were detected by phosphospecific antibodies. The bottom panel represents the Ser473 phosphorylation of endogenous PKB.
(5.6 MB EPS)

Figure S3 Effect of PH Domain Mutagenesis on PKB–PDK1 Docking (A) NIH3T3 cells were transfected either with GFP-PDK1 RRR alone or together with RFP-PKB as indicated. Upon 5 min PDGF stimulation, PH domain defective mutant GFP-PDK1 RRR did not translocate to the plasma membrane (the two-photon images of the donor). The lifetimes are presented in pseudo-colour scale ranging from 2.30 to 2.70 ns.
(B) The same experiment as in (A) but with GFP-PDK1 alone or cotransfected with RFP-PKB R25C was performed. Upon PDGF stimulation, PH domain defective RFP-PKB R25C did not translocate to the plasma membrane.
(C) The statistical analysis for experiments (A) and (B) is shown as box and whiskers plots. For both mutants, FRET efficiencies do not vary upon stimulation (*p* = 0.2).
(966 KB EPS)

Figure S4 Effect of Mutations on PKB–PDK1 Docking (A and B) GFP-tagged mutants of PDK1 PIF pocket (L155E) (A) and the phosphate-binding pocket (R131A) (B) were coexpressed with RFP-PKB.
(C) RFP-PKB AA, which cannot be phosphorylated on Thr308 and Ser473, was coexpressed with GFP-PDK1. Upon 5 min PDGF stimulation, GFP-PDK1 and RFP-PKB mutant constructs translocated to the plasma membrane in the same manner as the wild-type (wt) constructs (see Figure 1). The graph represents the distributions of the lifetimes of the cells under the following conditions: GFP-PDK1 wt or mutants alone (blue line) or GFP-PDK1 wt or mutant cotransfected with RFP-PKB wt or mutant prior (green line) or upon PDGF stimulation (orange line). The contribution of the pixels at the plasma membrane and cytoplasm are represented in red and light green, respectively.
(D) Statistical analysis of the FRET efficiencies is shown as box and whiskers plots. These mutations do not affect the interaction compared to the wt constructs.
(1.6 MB EPS)

Figure S5 Model for GFP and RFP Change in Distance upon GFP-PKB-RFP Change in Conformation (A) The kinase domain of PKB is shown in yellow ribbons with GFP and RFP in green and red ribbons, respectively. In cyan, PKB PH domain is shown in its “PH-in” conformation. The white dashed lines represent the linkages between the PKB domains and GFP or RFP.
(B) When PH domain moves to its “PH-out” conformation (dark blue), GFP moves to a new location. During this movement, the distance between GFP and RFP is roughly halved (distances shown in white lines are taken between the centres of domains or proteins).
(1.6 MB EPS)

Figure S6 Cotranslocation of RFP-PKB with GFP-PDK1 Shown by Confocal Imaging NIH3T3 cells were transfected with GFP-PDK1 and RFP-PKB. The cells were stimulated with PDGF for 5 min as indicated and fixed. Confocal images of the GFP-PDK1 and RFP-PKB were taken at mid-section using a Zeiss laser scanning confocal microscope (LSM 510), argon 458/488 laser with a ×63 oil Apochromat objective. A merge image was created to visualise in yellow the colocalisation of the two constructs at the plasma membrane upon PDGF stimulation.
(2.8 MB EPS)

### Accession Numbers

The GenBank (http://www.ncbi.nlm.nih.gov/Genbank) accession numbers for the genes and gene products discussed in this paper are mRFP (AF_506027), murine AKT1 (NM_009652), and construct pCMV5-EGFP-PDK1 (human PDK1, AF_017995). The Swiss-Prot (http://www.ebi.ac.uk/swissprot) database code for the homolog human Akt-2 kinase domain (PKBβ) is P31751.

## Abbreviations

Cy3: Cyan 3 dye
EGFP: enhanced green fluorescent protein
FLIM: fluorescence lifetime imaging microscopy
FRET: Förster resonance energy transfer
mRFP: monomeric red fluorescent protein
PDGF: platelet-derived growth factor
PDK1: 3-phosphoinositide–dependent protein kinase 1
PH: pleckstrin homology
PIF: PDK1 interacting fragment
PKB: protein kinase B
PtdIns: phosphoinositides
tau (or τ): refers to the lifetimes in the figures or in the text
